# Dynamic changes in injured myocardium, very early after acute myocardial infarction, quantified using T1 mapping cardiovascular magnetic resonance

**DOI:** 10.1186/s12968-018-0506-3

**Published:** 2018-12-20

**Authors:** Mohammad Alkhalil, Alessandra Borlotti, Giovanni Luigi De Maria, Lisa Gaughran, Jeremy Langrish, Andrew Lucking, Vanessa Ferreira, Rajesh K. Kharbanda, Adrian P. Banning, Keith M. Channon, Erica Dall’Armellina, Robin P. Choudhury

**Affiliations:** 1 Acute Vascular Imaging Centre, Radcliffe Department of Medicine, University of Oxford, Oxford, UK; 2 Oxford Heart Centre, NIHR Biomedical Research Centre, Oxford University Hospitals, Oxford, OX3 9DU UK; 3 Division of Cardiovascular Medicine, BHF Centre of Research Excellence, University of Oxford, Oxford, UK; 4 Division of Cardiovascular Medicine, University of Oxford Centre for Clinical Magnetic Resonance Research (OCMR), Oxford, UK

**Keywords:** STEMI, Area at risk, T1-mapping

## Abstract

**Background:**

It has recently been suggested that myocardial oedema follows a bimodal pattern early post ST-segment elevation myocardial infarction (STEMI). Yet, water content, quantified using tissue desiccation, did not return to normal values unlike oedema quantified by cardiovascular magnetic resonance (CMR) imaging. We studied the temporal changes in the *extent* and *intensity* of injured myocardium using T1-mapping technique within the first week after STEMI.

**Methods:**

A first group (*n* = 31) underwent 3 acute 3 T CMR scans (time-point (TP) < 3 h, 24 h and 6 days), including cine, native shortened modified look-locker inversion recovery T1 mapping, T2* mapping and late gadolinium enhancement (LGE). A second group (*n* = 17) had a single scan at 24 h with an additional T2-weighted sequence to assess the difference in the extent of area-at-risk (AAR) compared to T1-mapping.

**Results:**

The mean T1 relaxation time *value* within the AAR of the first group was reduced after 24 h (*P* < 0.001 for TP1 vs.TP2) and subsequently increased at 6 days (*P* = 0.041 for TP2 vs.TP3). However, the *extent* of AAR quantified using T1-mapping did not follow the same course, and no change was detected between TP1&TP2 (*P* = 1.0) but was between TP2 &TP3 (*P* = 0.019). In the second group, the extent of AAR was significantly larger on T1-mapping compared to T2-weighted (42 ± 15% vs. 39 ± 15%, *P* = 0.025). No change in LGE was detected while microvascular obstruction and intra-myocardial haemorrhage peaked at different time points within the first week of reperfusion.

**Conclusion:**

The intensity of oedema post-STEMI followed a bimodal pattern; while the extent of AAR did not track the same course. This discrepancy has implications for use of CMR in this context and may explain the previously reported disagreement between oedema quantified by imaging and tissue desiccation.

## Background

Following reperfusion treatment in patients with ST-segment elevation myocardial infarction (STEMI), there is an abnormal accumulation of water within the injured myocardium which, when depicted using cardiovascular magnetic resonance (CMR) imaging, defines ‘the area at risk’ (AAR) [1, 2]. The AAR represents tissue that could potentially be transformed into necrotic and infarcted tissue if the injured myocardium were not adequately reperfused; therefore quantifying AAR is important in calculating myocardial salvage index (MSI) [3, 4]. This index is an established measure for testing efficacy of therapies in acute STEMI, but relies on the assumption that AAR is stable post STEMI [3–5].

Recently, there has been debate over the stability of oedema /AAR immediately after reperfusion [6, 7]. Data from animal models, and subsequently humans, have suggested that oedema, as quantified on T2 weighted imaging and T2 mapping sequences, has a bimodal pattern [6, 7] The initial wave was attributed to reperfusion injury, which diminished after 24 h and was followed by a deferred wave which was related to tissue healing [6, 7]. The use of mapping techniques has added a new domain in measuring the *severity* of oedema beyond the anatomical *area* of oedema when quantifying the AAR [6, 7] In other words, quantification of water content becomes a consequence of the interaction of the *area* and the *severity* of oedema within AAR. The severity of oedematous reaction post reperfusion, quantified on T2 mapping, followed similar bimodal changes to the anatomical AAR, quantified as percentage of left ventricle (LV) on T2-weighted images [7]. Likewise, quantification of water content using a desiccation method followed bimodal wave, nonetheless, oedema did not normalise in 24 h, unlike T2 relaxation time [6]. This ‘residual’ water content is plausibly derived from persistent oedematous area, since its severity was reduced as measured using T2 mapping. Moreover, it raises the possibility that these previously applied CMR techniques may have failed to detect a relatively subtle persistent increase in water content after 24 h of reperfusion injury.

Native T1 mapping can characterise the injured myocardium and has been shown to characterise viable myocardium in the acute setting [8]. The increase in T1 value is secondary to an increase in myocardial free water content in response to an acute ischaemic insult [9, 10] More importantly, T1 mapping was demonstrated to be superior to T2 weighted imaging for the assessment of myocardial injury by identifying additional areas of abnormal myocardium in the setting of acute infarction and myocarditis [10, 11].

Here, we studied patients very early after reperfusion therapy and sought to investigate the temporal changes in AAR, quantified using a T1 mapping technique post STEMI. We hypothesised that bimodal changes in the *intensity* of oedematous reaction post ischemia/ reperfusion injury do not necessarily track with changes in the *extent* of the anatomical AAR (percentage of LV). This may provide a better reflection of actual changes in water content and would afford a better understanding of the application of CMR in assessments of acute myocardial injury.

## Methods

### Study population

Patients presenting with STEMI to the Oxford Heart Centre and who underwent primary percutaneous coronary intervention (PCI) were prospectively enrolled as part of the OxAMI Study [12, 13]. This was a pre-specified study within the OxAMI research programme, and patients were prospectively recruited. These data have not been reported in any other OxAMI published studies. STEMI was defined by ongoing chest pain for at least 30 min, associated with ST-segment elevation > 2 mm in at least two contiguous leads. Patients were excluded if the culprit vessel was not occluded (i.e. thrombolysis in myocardial infarction (TIMI) flow 0); if the anticipated symptoms to reperfusion duration was > 8 h; by the presence of severe hemodynamic instability, previous myocardial infarction, previous coronary artery bypass graft, pregnancy, severe renal impairment or contraindication to CMR including implanted pacemaker, defibrillator, or other metallic implanted devices and by claustrophobia.

**Fig. 1:**
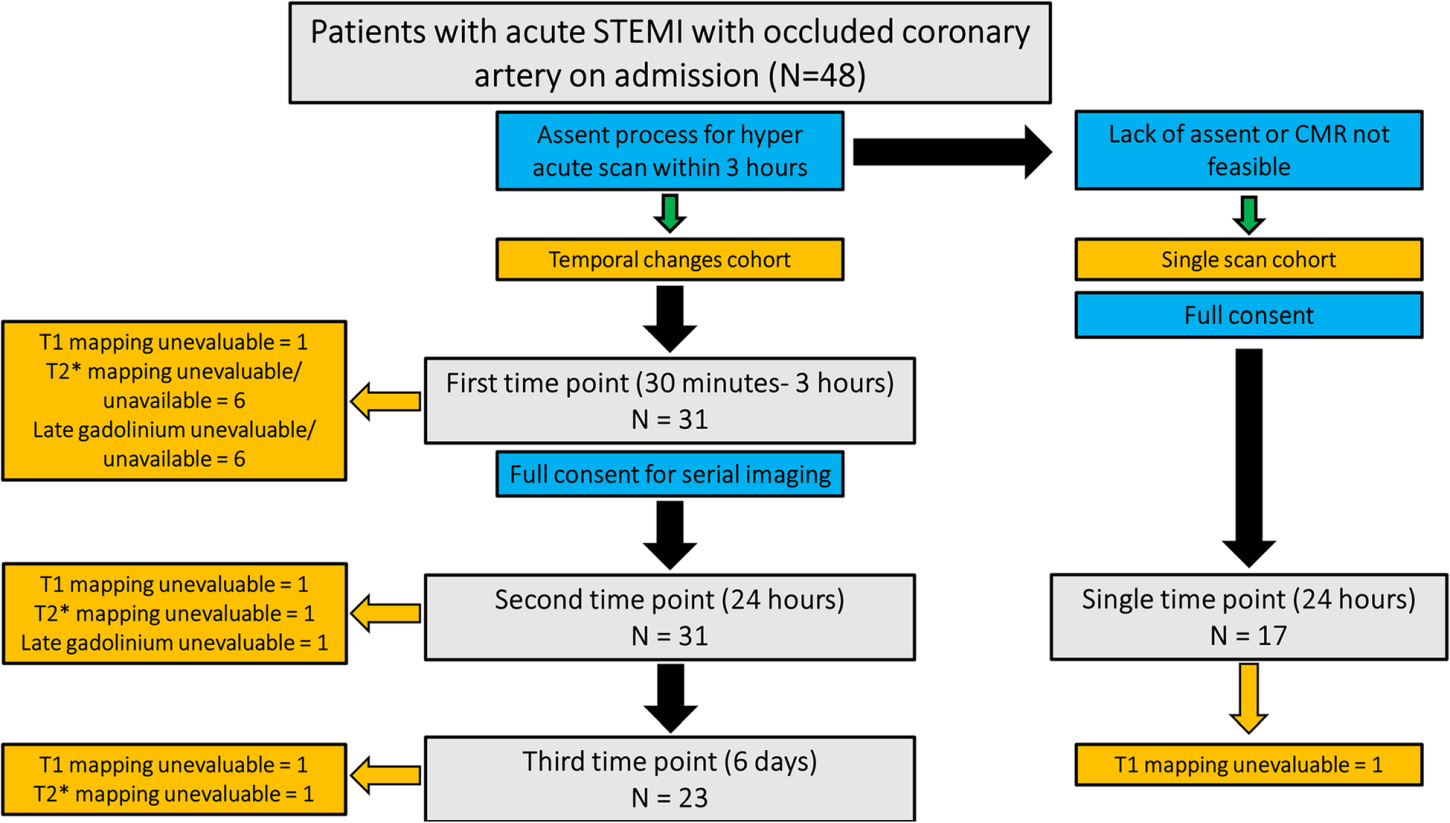
Study flow chart. Patients presenting with ST elevation myocardial infarction (STEMI) and occluded vessel were prospectively recruited to either temporal changes cohort or single scan cohort, depending on CMR feasibility within 3 h post stent implantation

Patients underwent serial imaging within 3 h of stent implantation, at 24 h, and after 5 to 7 days after the index presentation (*the temporal changes cohort*). The initial hyper-acute CMR scan was executed under an assent process [14] However, in those who did not assent but subsequently consented or when CMR was not feasible within the required timeframe (for logistic reasons), subjects were included in a *single scan cohort*, which was performed after 24 h from the presentation (Fig. 1).

### Cardiovascular magnetic resonance protocol

CMR was performed on a 3 Tesla scanner (MAGNETOM Verio, Siemens Healthineers, Erlangen, Germany). The scan protocol in *the temporal changes cohort* comprised balanced steady state free precession (bSSFP) for functional images, native Shortened Modified Look-Locker Inversion recovery (ShMOLLI) T1 mapping for AAR characterisation, [15] T2* mapping for quantification of intramyocardial haemorrhage (IMH) and late gadolinium enhancement (LGE). The *single scan cohort* protocol was identical but additionally a T2-weighted (T2-prepared bSSFP) sequence was obtained to compare anatomical AAR between acquired T1 mapping and T2-weighted imaging.

T1 maps, T2* maps, late gadolinium enhancement (LGE) and bSSFP short axis images were acquired on matching short-axis slices covering the entire LV; only 3 matching short axis slices were acquired for T1 and T2* mapping in the hyper-acute phase targeting the regional wall motion abnormalities as assessed on functional images.

Typical acquisition parameters for bSSFP retrospectively gated cine images were TE / TR =1.4/3.2 ms; flip angle 50°; voxel size: 2.4 × 1.8 × 8.0 mm. To shorten the hyperacute scan, the cine sequence at time point (TP)1 were acquired in a single breath hold using real time electrocardiogram (ECG)-triggered and at lower resolution (160 × 72) compared to other time points (224 × 137).

T2W was performed using a T2-prep-bSSFP single shot sequence with surface coil correction (TE/TR = 1/4.1 msec; effective TE = 60 msec; flip angle 90°; voxel size: 2.1 × 1.6 × 8.0 mm).

ShMOLLI T1 maps were generated from 5 to 7 bSSFP images with variable inversion preparation S2 time as described previously [15]. Typical acquisition parameters were: TE/TR = 1.07/2.14 msec, flip angle = 35°, FOV = 340 × 255 mm, matrix size = 192 × 144, 107 phase encoding steps, actual experimental voxel size = 1.8 × 1.8 × 8 mm, interpolated reconstructed voxel size = 0.9 × 0.9 × 8 mm, GRAPPA = 2, 24 reference lines, cardiac delay time TD = 500 msec and 206 msec acquisition time for single image, phase partial Fourier 6/8.

T2* maps were obtained using a gradient echo sequence. Typical imaging parameters were: TR 600 ms, echo numbers (*n* = 5), TE 22.14 ms, FOV = 340 × 225 mm, bandwidth 260 Hz/Px, matrix = 192 × 144, voxel size = 1.8 × 1.8 × 3.0 mm, flip angle 20°.

LGE was performed with a T1- weighted segmented inversion recovery gradient echo-phase sensitive-inversion recovery (GRE_PSIR) sequence (TE/TR = 2.5 msec/5 msec, bandwidth 781 Hz/Px, matrix = 256 × 167, voxel size = 1.8 × 1.4 × 8.0 mm, flip angle 40°). LGE images were collected 10–15 min after the administration of 0.1 mmol/kg contrast agent (Dotarem, Guerbet, Villepinte, France) [8]. The inversion time was adjusted for optimal nulling of remote normal myocardium.

### CMR imaging analysis

Cvi42 image analysis software (Circle Cardiovascular Imaging Inc., Calgary, Canada) was used for image analysis by two experienced operators in CMR image analysis, blinded to CMR timing and measurements of the same patient across three time points. LV volumes and ejection fraction (EF) were assessed from cine bSSFP images. AAR on T1 mapping/ T2-weighted was identified similarly to previous reports using a signal intensity threshold of 2SD above the mean intensity of remote reference region of interest (ROI) placed 180 degree opposite to the injured myocardium with no visible regional wall abnormalities or infarction (assessed by inspecting corresponding cine and LGE images, respectively) [16, 17]. T1 values of the delineated area were subsequently averaged to assess the severity of oedematous reaction, additionally, this area was measured as a percentage of the LV mass. T1 values were calculated for each patient averaging T1 values of AAR on the 3 matching short-axis slices and to track changes in AAR over time, only the same anatomical slices in all acquired images of consecutive time points were compared serially.

T1 core was defined as a central area within the injured myocardium with T1 value less than 2SD below the mean T1 value of the periphery of AAR as previously described [18]. Since T1 core affects the T1 values inside the AAR, T1 value was also averaged in the injured myocardium excluding the core.

To quantify injured myocardium, as depicted by LGE, signal intensity threshold was set at 5 standard deviations above the remote reference myocardium [13]. When present, T1 core and microvascular obstruction (MVO) were included in the measurements of AAR and LGE, respectively. The LV MVO percentage fraction was quantified by manual delineation of the hypointense areas within the LGE region [19] IMHas defined on T2* maps as a hypointense area within the injured myocardium having a mean signal intensity 2SD below the signal intensity of the periphery of oedematous region and a mean values < 20 ms and was manually delineated to yield percentage of LV.

MSI was calculated from 24 h scan using T1 map to derive AAR as previously described [8].

### Statistical analysis

Normality of distribution was assessed using the Shapiro-Wilk test. All variables were expressed as mean and ± standard deviation or as median accompanied by interquartile range (IQR) based on normality distribution. Frequencies comparisons were made using Chi square test or Fisher’s exact test, as appropriate while continuous variables were compared by using unpaired T test or Mann-Witney U test where appropriate. Paired t test was used to compare AAR using T1 mapping and T2-weighted in the single scan cohort. To track changes of T1 values over time taking into account repeated measures, generalised mixed model was conducted with time and haemorrhage as fixed effects and subject identification as random effect. This was done given that haemorrhage is known to affect magnetic resonance properties [20]. Post hoc multiple pairwise comparisons were performed with Bonferroni adjustment. All statistical analyses were performed using SPSS 22.0 (Statistical Package for the Social Sciences, International Business Machines, Inc., Armonk, New York, USA) and a *P* value < 0.05 was considered statistically significant.

## Results

A total of 48 patients were included in this study with total of 102 CMR exams. Of these, 31 patients underwent the 3 h hyper-acute CMR scan, followed by 24 h (31 patients) and 6 days (23 patients) CMR scans. These patients formed the temporal changes cohort. The remaining 17 patients underwent a single CMR scan at 24 h (Fig. 1). Two patients (1 from each cohort) had unevaluable T1 mapping and were excluded from the analysis. The clinical and CMR characteristics of the remaining 46 patients are shown in Tables 1 and 2. Importantly, there were no differences in clinical or procedural characteristics between two cohorts and, similarly, their CMR characteristics, spanning the whole LV, were comparable.

**Table 1:** Clinical characteristics of included patients, stratified according to the number of acquired CMR scans

Patients characteristics	Temporal change cohort (*N* = 30)	Single scan cohort (*N* = 16)	*P* value
Age^(a)^	60 ± 11	56 ± 8	0.26
Male gender^(c)^	27 (90%)	16 (100%)	0.54
Hypertension^(c)^	11 (37%)	4 (25%)	0.42
Dyslipidaemia^(c)^	10 (33%)	6 (38%)	0.77
Active smoking^(c)^	9 (30%)	2 (13%)	0.19
Diabetes^(c)^	2 (7%)	1 (7%)	1.0
Ischaemia time^(b)^ (mins)	188 (149–292)	165 (102–251)	0.21
Systolic pressure^(a)^	124 ± 27	131 ± 24	0.40
Diastolic pressure^(a)^	75 ± 16	80 ± 12	0.29
Preloading clopidogrel^(c)^	21 (70%)	10 (63%)	0.61
Anterior infarct^(c)^	8 (27%)	8 (50%)	0.19
Thrombectomy use^(c)^	19 (63%)	8 (50%)	0.38
Glycoprotein IIb/IIIa inhibitors^(c)^	5 (17%)	3 (19%)	0.86
Stent length^(b)^ (mm)	28 (22–32)	26 (20–30)	0.49
Stent diameter^(b)^ (mm)	3.5 (3.4–4)	3.5 (3 4)	0.27
Final TIMI III flow^(c)^	25 (83%)	13 (81%)	1.0

Data are presented as ^(a)^ mean ± SD, ^(b)^ median & IQR, ^(c)^ total number and proportion, TIMI (thrombolysis in myocardial infarction)

**Table 2:** 24 h CMR characteristics of included patients in each cohort

Patients characteristics	Temporal change cohort (*N* = 30)	Single scan cohort (*N* = 16)	*P* value
End Diastolic Volume (EDV)^(a)^ (ml)	168 ± 38	177 ± 36	0.42
End Systolic Volume (ESV)^(a)^ (ml)	88 ± 33	85 ± 24	0.73
Ejection Fraction (EF)^(a)^	48 ± 11	52 ± 8	0.27
Area At Risk (AAR)^(a)^ (using T1 mapping)	38.5 ± 15	38.0 ± 14	0.92
Late Gadolinium Enhancement (LGE) (LV %)^(b)^	21 (17–33)	23 (13–32)	0.72
Microvascular Obstruction (MVO) (LV %)^(b)^	1.0 (0–6.0)	0.7 (0–4.3)	0.76
Incidence of MVO^(c)^	15 (50%)	8 (50%)	1.0
Myocardial Salvage Index (MSI) (LV %)^(b)^	34 (22–57)	40 (30–53)	0.45

Parameter was calculated from all LV slices. Data are presented as (a) mean ±SD, (b) median & IQR, (c) total number and proportion

CMR scans were performed at 1.6 h (1.2–2.4), 25.5 (23.8–26.0) hours and 6.5 (6, 7) days in the temporal changes cohort, compared to a median of 23.5 h (22–28.8) in the single scan cohort (*P* = 0.51 for difference with second time point in temporal changes cohort). All hyper acute scans were performed between 30 min and 3 h after placing a stent in the culprit artery. No significant arrhythmias were detected during scans acquisition.

### Changes in T1 relaxation time over time

**Fig. 2:**
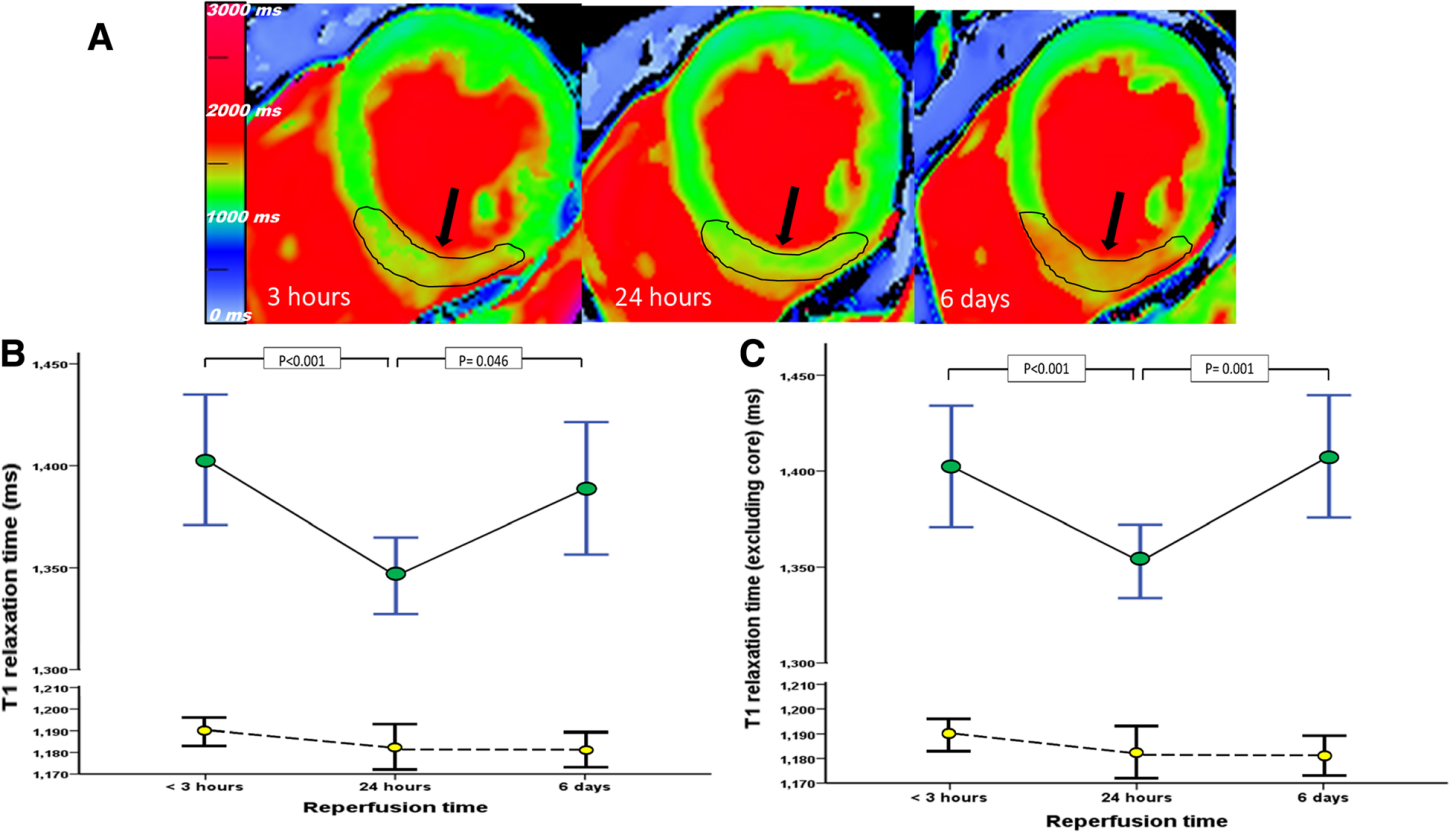
Bimodal changes of T1 relaxation time. Panel **a** short-axis T1 maps in a patient with inferior ischaemic injury (black arrow) acquired at three time points post STEMI presentation. The magnitude of change in native T1 value within the area at risk (AAR) (contoured in black) is illustrated against a colour scale map. Panel **b** T1 relaxation time showed significant attenuation of oedematous reaction in the injured myocardium after 24 h and returns to previous level at 6 days (blue lines representing mean +/− 2 standard error). Changes within remote myocardium over time are illustrated in black lines (mean +/− 2 standard error). Panel **c** excluding central area with low T1 values (T1-core), T1 relaxation time was compared serially. The deferred wave of oedematous reaction was more prominent with significant difference between 24 h and 6 days (blue lines representing mean +/− 2 standard error)

The mean T1 relaxation time within the injured myocardium was significantly higher than remote myocardium at the hyper acute time point (1404 ± 76 ms vs. 1189 ± 16 ms, *P* < 0.001). T1 relaxation time showed significant reduction at 24 h after reperfusion therapy at the second time point (*P* < 0.001). Subsequently, the T1 value rose to a value similar to that at TP1 and was significantly different from the 24 h scan (*P* = 0.041) (Table 3) (Fig. 2a and b).

**Table 3:** Dynamic changes of injured and remote myocardium within the first week following STEMI

Studied myocardium	Reperfusion time			Pairwise comparison (*P* value)		
	< 3 h	24 h	6 days	A*	B*	C*
T1 relaxation time (inc core) ^(b)^*ms*	1404 ± 76	1345 ± 49	1384 ± 77	< 0.001	0.81	0.046
T1 relaxation time (remote) ^(b)^ *ms*	1189 ± 16	1182 ± 27	1181 ± 19	0.14	0.004	1.0
T1 relaxation time (exc core) ^(b)^*ms*	1406 ± 71	1353 ± 55	1398 ± 67	< 0.001	1.0	0.001
Injured myocardium (AAR, LV %) on T1 map^(b)^	37 ± 11	38 ± 11	42 ± 9	1.0	0.001	0.019
T1 core area^(b)^ (mm^2^)	0.15 ± 1.14	1.1 ± 1.14	0.84 ± 1.15	< 0.001	0.028	0.99
T1 core prevalence^(c), a^	4 (13%)	15 (50%)	9 (41%)	–	–	–

^a^*P* value is not presented because generalised linear mixed-effects model does not support analysis for categorical data. Data are presented as ^(b)^ mean ± SD, ^(c)^ total number and proportion. * A (comparison between < 3 & 24 h), B (comparison between < 3 h & 6 days), and C (comparison between 24 h & 6 days)

Unlike the injured myocardium, the remote myocardium had a trend towards reduction in T1 relaxation time over time, nonetheless the absolute difference across the 3 time points was nominal (< 10 ms). There was no significant difference in T1 value between 3 h and 24 h (*P* = 0.14), and similarly no significant difference between 24 h and 6 days CMR scan (P = 0.1). However, a significant difference was detected when comparing first and third time points (*P* = 0.004). All remote T1 values were similar to those previously reported in healthy subjects scanned in our department [15].

### Changes in extent of oedema

**Fig. 3:**
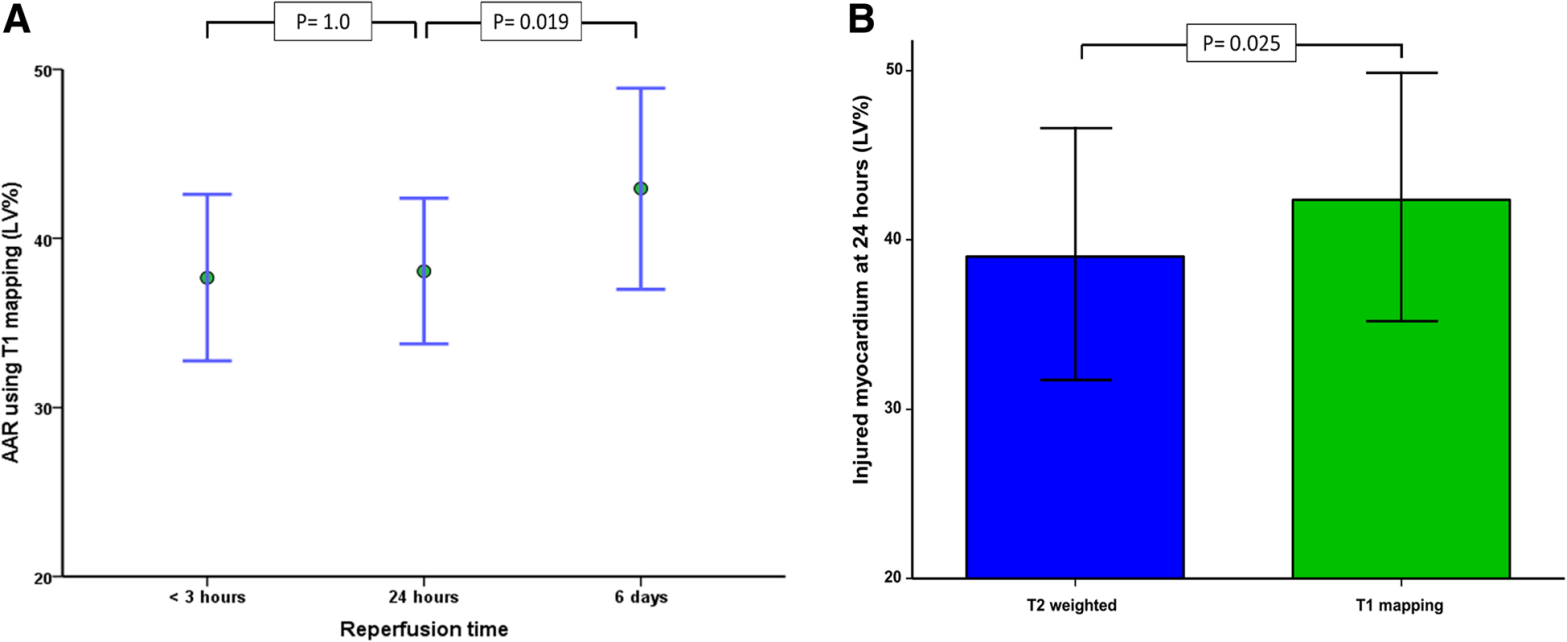
Changes in extent of oedema. Panel **a** AAR, quantified using T1 mapping sequence, did not undergo significant changes within 24 h. There was significant increase in extent of oedema at 6 days mirroring increase in T1 relaxation time (mean +/− 2 standard error). Panel **b** pairwise comparison between injured myocardium in T2-weighted and T1 mapping (mean +/− 2 standard error)

Injured myocardium as identified on T1 mapping (AAR) was not solely analysed for changes in the *intensity* of the oedema (illustrated above). The changes in the *anatomical area* or the *extent* of injured myocardium was also studied and showed no significant change at 24 h, compared to 3 h (*P* = 1.0). Subsequently, the extent of AAR by T1 mapping increased when compared with the second CMR time point (*P* = 0.019) (Table 3) (Fig. 3a).

The extent of oedema was compared using T1 mapping and T2 weighted sequences in the single scan cohort. Interestingly, AAR differed significantly when assessed using T1 mapping versus T2-weighted, (39 ± 15% by T2W vs. 42 ± 15% by T1 map, *P* = 0.025) (Fig. 3b).

### Changes of T1 core and its impact on bimodal changes

The bimodal reaction of injured myocardium was similar, if not more pronounced, when excluding area of T1 core from the analysis. This was particularly notable for the difference in T1 relaxation time between the second and third time points (*P* = 0.001) (Table 3 & Fig. 2c).

T1 core area increased significantly after 24 h following STEMI (*P* < 0.001) and subsequently stayed the same at 6 days post STEMI (*P* = 0.99). Similarly, the incidence of T1 core followed similar pattern and 50% of subjects developed T1 core within the injured myocardium at 24 h compared to only 13% at the hyper acute scanning.

### Changes in late gadolinium enhancement, microvascular obstruction and intramyocardial hemorrhage

Enhanced myocardium quantified on LGE sequence was comparable with no significant differences across three time points (Table 4).

**Table 4:** Dynamic changes of LGE, MVO and IMH within the first week following STEMI

	Time from reperfusion			Pairwise comparison (*P* value)		
	< 3 h	24 h	6 days	A*	B*	C*
Ejection fraction (EF) (%)	49 ± 11	48 ± 11	51 ± 9	0.66	0.45	0.21
End Diastolic Volume (EDV) (*ml)*	162 ± 33	168 ± 33	177 ± 29	0.11	0.001	0.015
End Systolic Volume (ESV)*(ml)*	83 ± 27	89 ± 27	89 ± 24	0.085	0.057	1.0
Late gadolinium enhancement (LGE) (LV %)	27 ± 16	26 ± 16	27 ± 19	1.0	0.99	0.87
Microvascular obstruction (MVO) (LV %)	4.0 ± 4.9	4.1 ± 6.0	2.4 ± 3.4	1.0	0.02	0.011
MVO prevalence (n, %)	11 (44%)	17 (56%)	12 (55%)	–	–	–
Intra-myocardial haemorrhage (IMH) *mm*^*2*^	0.7 ± 1.6	1.4 ± 1.6	1.1 ± 1.4	0.012	0.46	0.69
IMH prevalence (n, %)	9 (36%)	14 (47%)	10 (45%)	–	–	–

All data are presented as mean ± SD, * A (comparison between < 3 & 24 h), B (comparison between < 3 h & 6 days), and C (comparison between 24 h & 6 days)

**Fig. 4:**
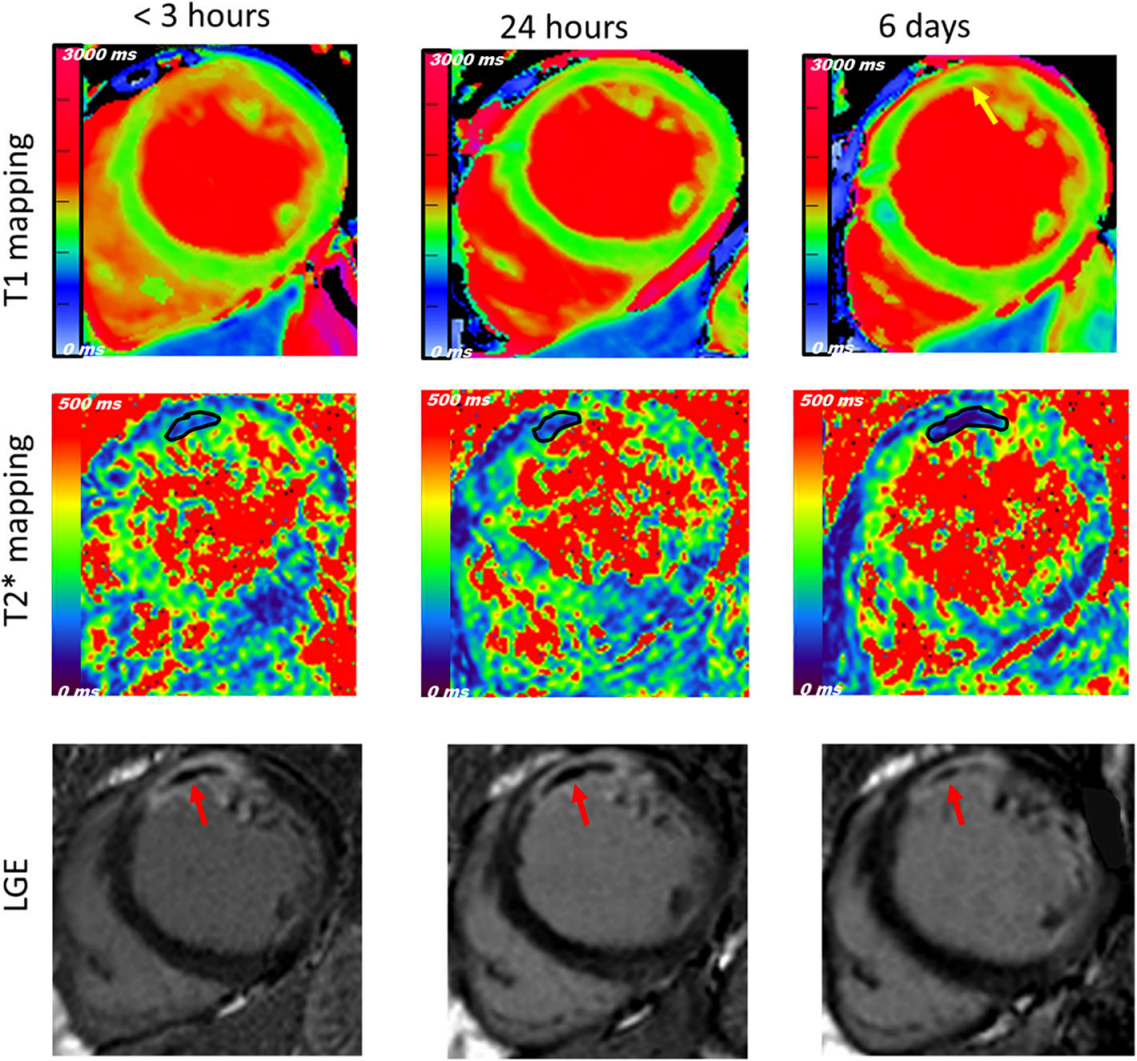
Changes in T1 core, LGE, MVO and IMH. Development of T1 core (yellow arrow), area of low T1 value within injured myocardium that progressed over time. The extent of late gadolinium enhancement (LGE) was comparable across time points with early peak of microvascular obstruction (MVO) (red arrows) compared to more lagged peak of intramyocardial haemorrhage (IMH) (contoured in black)

MVO, on the other hand, showed dynamic changes within the first week. MVO peaked within the first 24 h with no difference between the first two time points (*P* = 1.0). Subsequently, MVO was significantly reduced (TP2 vs. TP3, *P* = 0.011) and the reduction was also significant when comparing TP1 vs TP3 (*P* = 0.02) (Table 4) (Fig. 4).

IMH followed a different pattern and increased significantly after 24 h following STEMI (TP1 vs TP2, *P* = 0.012). IMH remained comparable between 6 days CMR scanning and both 3 h (*P* = 0.46) and 24 h CMR scanning (*P* = 0.69) (Table 4) (Fig. 4).

## Discussion

This is the first study to investigate the dynamic changes of highly sensitive T1 mapping immediately post STEMI. The main findings of this study can be summarised as follows: (1) after reperfusion therapy in patients presenting with STEMI, the intensity of oedematous response assessed using T1 mapping on 3 T CMR was not stable and followed bimodal pattern; (2) this bimodal reaction was still evident when excluding ‘T1 core’, which increased in area and, possibly, in prevalence, over time; (3) the *extent* of the anatomical AAR did not follow the same course as the T1 *value* and was not reduced after 24 h; and (4) LGE remained constant within the first week, unlike both MVO and IMH which peaked within the first 7 days.

Accurate quantification of myocardial area at risk is essential in calculating MSI, which has been used to assess efficacy of applied therapies in patients presenting with STEMI [5]. Timing of the ‘acute’ CMR scanning following acute myocardial infarction has lacked consistency based on the assumption that AAR assessed by standard T2W techniques is stable over the first one week and its quantification could be measured retrospectively, in part supported by our own earlier observations [21]. Recently, however concerns have been raised about the stability of the AAR and evidence from both animal and human studies have challenged this concept, suggesting that changes in myocardial oedema followed a bimodal pattern [6, 7] Carrick et al. [20, 22] proposed that deoxyhaemoglobin was exclusively responsible for the decrease in T2 relaxation time after 24 h. They suggested that these dynamic changes were a pure CMR phenomenon and did not reflect real changes within AAR [20, 22]. They proposed that changes within the AAR were only apparent in STEMI population with evidence of IMH on CMR. However, data from our study demonstrated that changes in the intensity of oedema post-STEMI followed a bimodal pattern. The extremely early (median 1.6 h) CMR time point adopted in the current study showed that the initial peak took place within the first three hours, a hyper-acute time point that was not previously available [20].

Recently, Fernández-Jiménez et al. [7] reported bimodal changes in humans replicating previously reported findings in an animal model [6]. Using multi-detector computed tomography (MDCT) to quantify AAR at the time of vessel occlusion, they noted that T2W had, in fact, underestimated AAR at 24 h following STEMI in the animal model. Furthermore, the same group reported that water content quantified on desiccation did not normalise, in contrast to the AAR delineated using T2W sequence in addition to T2 relaxation times. Both extent and severity of injured myocardium were reduced after 24 h to match pre-infarct measurements [6]. In other words, it appeared that the earlier CMR techniques were not sufficiently sensitive to detect residual oedema. We demonstrate here, for the first time, that changes in the anatomical area of oedema did not follow the same course as the *intensity* of the oedematous reaction measured using mapping technique (T1 relaxation time). This implies that the water content of a given volume of myocardium can change, somewhat like a sponge taking water, where changes in water content do not necessitate changes in the overall volume of the sponge. Moreover, we compared the difference between anatomical AAR determined by T1 mapping and T2W imaging and demonstrated that T2W underestimated the injured myocardium 24 h after STEMI. Taking all these observations together, these findings are consistent with the residual water content identified using desiccation method, which can be considered a gold standard. More importantly, it highlights the stability of AAR measured using T1 mapping and would allow the calculation of MSI within the first week.

An area with low T1 relaxation time, termed the ‘T1 core’, within injured myocardium showed some impact on the dynamic changes of AAR. T1 core has been shown to be a significant imaging biomarker to characterise infarcted myocardium and to predict future cardiovascular events [18]. We demonstrated that this area increases over time post STEMI and possibly in prevalence. Therefore, it would be anticipated that it may reduce the T1 relaxation time and possibly ameliorate the bimodal reaction by decreasing the “intensity” of oedematous reaction after 24 h. The significance relevance of expanded T1 core over time may need further assessments in prospective studies.

In this study, patients underwent LGE and T2* mapping sequences at three time points to assess if dynamic changes occur with the first week. Enhanced myocardium showed minimal changes overtime, unlike MVO which appears to peak within the first 24 h. Experimental studies using CMR coupled with blood flow measurement employing microspheres have demonstrated a similar course to our data [23]. Moreover, Robbers et al. [19] demonstrated that areas of MVO lack vascular integrity with higher number of vessels in the gadolinium enhanced myocardium compared to contrast-devoid core [19]. IMH, on the other hand, appears to peak at different timing from MVO. Our study demonstrated a significant increase in IMH after 24 h, which did not parallel MVO changes. Previous reports have illustrated that IMH peaks after 3 days, a time point that is not available in our study [7, 20]. Our data extend these findings by comparing MVO & IMH changes at extremely early time points. While MVO was already established by 3 h, IMH required longer period to reach its peak and lagged behind the no-reflow phenomenon. This adds additional insights to the intricate relationship between the extravasation of erythrocytes with IMH presence and MVO [19, 24] The early presence of IMH in some individuals may also reflect a more severe ischaemic insult or even worse reperfusion injury [19, 24, 25].

Our study in vivo in humans lacks histological / tissue validation to confirm findings using T1 mapping technique. We did not compare T2-weighted and T1 mapping at the hyper acute time point given the time constraint to perform lengthy CMR scan immediately after STEMI in potentially unstable patients. However, we validated our finding using a prospective cohort employing both T2-weighted imaging and T1 mapping sequences to measure AAR in humans. In addition, previous reports have demonstrated that T2-weighted imaging may underestimate AAR when compared to desiccation or MDCT [6, 7]. Finally, our cohort included both left anterior descending (LAD) and non-LAD infarcts and yet this did not influence the dynamic changes within the injured myocardium [7].

## Conclusions

The intensity of the oedematous reaction post STEMI follows a bimodal pattern; while the extent of AAR does not track the same course and was stable over the first week. This discrepancy has important implications for use of CMR in this context and may explain the previously reported disagreement between oedema quantified by imaging and by tissue desiccation. We conclude that anatomical area at risk may best be quantified using T1 mapping, undertaken in the first week after reperfusion.

## Abbreviations

AAR: Area at risk
bSSFP: Balanced steady state free precession
CMR: Cardiovascular magnetic resonance
ECG: Electrocardiogram
EDV: End diastolic volume
EF: Ejection fraction
ESV: End systolic volume
IMH: Intra-myocardial haemorrhage
IQR: Interquartile range
LAD: Left anterior descending coronary artery
LGE: Late gadolinium enhancement
LV: Left ventricle/left ventricular
MDCT: Multi-detector computed tomography
MSI: Myocardial salvage index
MVO: Microvascular obstruction
OxAMI: Oxford for Acute Myocardial Infarction
PCI: Percutaneous coronary intervention
ROI: Region of interest
ShMOLLI: Shortened Modified Look-Locker Inversion recovery
STEMI: ST-segment elevation myocardial infarction
TIMI: Thrombolysis in myocardial infarction
TP: Time point
